# Reducing unnecessary hospital days to improve quality of care through physician accountability: a cluster randomised trial

**DOI:** 10.1186/1472-6963-13-14

**Published:** 2013-01-10

**Authors:** Caterina Caminiti, Tiziana Meschi, Luca Braglia, Francesca Diodati, Elisa Iezzi, Barbara Marcomini, Antonio Nouvenne, Eliana Palermo, Beatrice Prati, Tania Schianchi, Loris Borghi

**Affiliations:** 1 Research and Innovation Unit, University Hospital of Parma, Via Gramsci 14, Parma, 43126, Italy; 2 Critical Long-Term Care Unit, University Hospital of Parma, Via Gramsci 14, Parma, 43126, Italy

**Keywords:** Unnecessary hospital days, Audit, Physician accountability, Cluster randomised trial, Quality of care

## Abstract

**Background:**

Over 20% of hospital bed use is inappropriate, implying a waste of resources and the increase of patient iatrogenic risk.

**Methods:**

This is a cluster, pragmatic, randomised controlled trial, carried out in a large University Hospital of Northern Italy, aiming to evaluate the effect of a strategy to reduce unnecessary hospital days. The primary outcome was the percentage of patient-days compatible with discharge. Among secondary objectives, to describe the strategy’s effect in the long-term, as well as on hospital readmissions, considered to be a marker of the quality of hospital care. The 12 medical wards with the longest length of stay participated. Effectiveness was measured at the individual level on 3498 eligible patients during monthly index days. Patients admitted or discharged on index days, or with stay >90 days, were excluded. All ward staff was blinded to the index days, while staff in the control arm and data analysts were blinded to the trial’s objectives and interventions. The strategy comprised the distribution to physicians of the list of their patients whose hospital stay was compatible with discharge according to a validated Delay Tool, and of physician length of stay profiles, followed by audits managed autonomously by the physicians of the ward.

**Results:**

During the 12 months of data collection, over 50% of patient-days were judged to be compatible with discharge. Delays were mainly due to problems with activities under medical staff control. Multivariate analysis considering clustering showed that the strategy reduced patient-days compatible with discharge by 16% in the intervention vs control group, (OR=0.841; 95% CI, 0.735 to 0.963; P=0.012). Follow-up at 1 year did not yield a statistically significant difference between the percentages of patient-days judged to be compatible with discharge between the two arms (OR=0.818; 95% CI, 0.476 to 1.405; P=0.47). There was no significant difference in 30-day readmission and mortality rates for all eligible patients (N=3498) between the two arms.

**Conclusions:**

Results indicate that a strategy, involving physician direct accountability, can reduce unnecessary hospital days. Relatively simple interventions, like the one assessed in this study, should be implemented in all hospitals with excessive lengths of stay, since unnecessary prolongation may be harmful to patients.

**Trial registration:**

ClinicalTrials.gov, identifier NCT01422811.

## Background

Research conducted in various countries shows that a substantial proportion of hospital days is devoted to nonacute care that could be delivered outside of an inpatient setting. A review of studies aimed at identifying the extent of inappropriate bed use concludes that, despite large variation, inappropriateness is greater than 20% across a wide variety of settings [1]. Besides implying a waste of economic and human resources, unneeded prolonged hospital stay can be detrimental to patients, as they are exposed to risks of iatrogenic complications that may result in substantial morbidity and mortality [2]. For older patients, moreover, longer hospital stays can be particularly harmful, increasing the likelyhood of functional decline with consequent negative impact on quality of life [3].

Observational studies investigating the reasons for excessive length of stay (LOS) highlight issues related to access to community services, delays in medical care, as well as the crucial role of clinician attitude [4-6].

Various interventions have been adopted to reduce unnecessary LOS, including discharge planning and programs favoring transfer to community services, care pathways, reminders to sensitise clinicians, periodical audits to identify and act upon reasons for delays, use of checklists for admission planning, identification of motivated reference physicians. Studies evaluating such interventions however are mostly observational [7-9], or are randomised trials but assess the impact of single interventions on specific conditions or procedures [10-13]. When the protocol of this study was developed, no commonly accepted strategy aimed at the reduction of avoidable LOS existed.

Since 1998, the analysis of administrative data conducted at the University Hospital of Parma shows that the number of hospital days in acute wards is significantly higher than the mean DRG-specific values (Diagnosis-Related Groups) recorded in health care institutions of the Emilia-Romagna Region. This information can be easily derived from the annual reports on the performance of all regional hospitals, which the Regional Health Trust makes publicly available on its website [14]. Despite the introduction of different measures [15,16], the trend was still increasing. These findings emphasised the urgency to devise an effective strategy aimed at the reduction of avoidable hospital days at our institution.

We decided to conduct at our hospital a two-arm cluster randomised trial, with the aim to evaluate the effect of a multicomponent strategy, of which audit and feedback (A/F) was the core element, intended to reduce excessive and avoidable days of hospitalization. A/F are widely used interventions to improve professional practice, either alone or as a component of multifaceted quality improvement interventions, based on the belief that healthcare professionals are prompted to modify their practice when given performance feedback showing that their clinical practice is inconsistent with a desirable target. In fact, a systematic review of randomized trials reporting objectively measured outcomes has shown that A/F can be effective in improving professional practice, particularly when baseline adherence to recommended practice is low [17]. To ensure generalisability and minimise contamination bias, a pragmatic trial with wards as random assignment units was performed, whereas outcomes were measured at the patient level.

This study is reported in accordance with the indications provided in the CONSORT (CONsolidated Standards Of Reporting Trials) statement [18], in particular in its extension to cluster randomised trials [19] and with the SQUIRE (Standards for QUality Improvement Reporting Excellence) guidelines for quality improvement reporting [20].

## Methods

### Setting and participants

The University Hospital of Parma is one of the largest Italian health care facilities, with full-time core residency training programs in medicine and surgery, with 1267 beds and over 52000 admissions/year. The General Medicine and Geriatrics units mainly admit patients from the Emergency Room (90% of cases) and cover 14% of overall admissions. 6% of patients are transferred to the long-term units. The hospital works in strict cooperation with community health and social services.

The 12 hospital wards with the longest LOS participated in the study; these are all medical wards, and include eight general medicine, two geriatrics and two long-term care units. Although long-term care units are expected to care for patients requiring extended hospitalisations, these wards were included in the study because they exhibited longer LOS compared with similar wards in other institutions of the region. The Directors of the participating wards, or a delegate, acted as reference physicians for the project; the study was presented to them as a quality-improvement project, without emphasizing its aims and research methodology.

### Objectives and outcomes

The primary objective was to evaluate the effect of a strategy aimed to reduce unnecessary hospital days over a 12-month period. The main efficacy endpoint was the percentage of patient-days compatible with discharge measured on an index day. To identify such days we employed the Delay Tool developed by Carey et al. [5] (see Data Collection).

Secondary objectives were:

1. To describe the strategy’s effect in the long-term, measured in both arms one year after the end of implementation, on an index day.

2. To analyse the strategy’s effect in terms of overall length of stay for subjects included in the investigation. Information on each patient’s length of stay was retrieved from routinely collected administrative data.

3. To verify whether the strategy’s effect was greater for specific causes of delay, or generally distributed, according to the information gathered with the Delay Tool.

4. To compare readmission and mortality rates in the year of implementation between the two arms. Readmission rate is defined as the number of subjects included in the investigation who experienced unintended, acute readmission in any ward of any hospital, within 30-days of discharge from the day of admission, divided by the total number of patients included in the study. Mortality rate is defined as the number of subjects included in the investigation who died within 30-days of discharge from the day of admission, divided by the total number of patients included in the study [21]. All objectives pertain to the individual level, considering clustering.

### Study design

This is a cluster, parallel group, randomised trial, where the tested strategy is targeted at the wards (the units of random assignment) and the effectiveness is measured at the individual level, with the patient-day as unit of analysis. The strategy was implemented from February 2008 to February 2009, excluding August 2008. It was decided not to include August, because the reduction of staff and bed capacity during the holiday season, with consequent ward reorganisation (e.g. temporary merging of wards), made this month non-homogeneous with respect to other periods. One year after the end of implementation (March 2010) the long-term effect was measured in both arms. Data analysis was completed in February 2011.

### Randomisation and masking

The 12 wards were assigned by equal randomisation (1:1) to an intervention arm, where the strategy was implemented, or to a control arm, where assessment only was introduced. Randomisation was stratified according to ward type, which was possible because of the coincidental even number of wards for each type.

Centralised randomisation with computer sequence generation, for ward allocation and identification of the index days, was performed with blinding by a statistician. The sequence was concealed until interventions were assigned. Staff of all participating wards was blinded to the index days for data collection, and staff in the wards of the control arm, as well as data analysts, were blinded to the trial’s objectives and interventions.

### Intervention

The strategy was intended to motivate individual physicians to adopt more efficient practice patterns. It comprised two integrated components:

1. Distribution of two monthly reports, one consisting in the list of patients who, through data collection performed on the index days with the Delay Tool (see Data Collection), were classified to be present on the ward although their clinical status was considered compatible with discharge; the other featuring individual length of stay profiles for each physician operating in the intervention arm (information taken from administrative data), allowing them to compare their own performance with that of the rest of the medical staff, similarly to the approach described by Lagoe et al. [8].

2. Audits performed by professionals of each ward of the intervention arm designed to discuss cases judged to be compatible with discharge. The organisation of this work, as well as the identification and implementation of improvement measures, were left to the wards, without any interference from the project team.

The study was reviewed by the Institutional Ethics Committee of the University Hospital of Parma, and conducted in accordance with the protocol. Because this study aimed to test the effect of quality improvement measures, with no direct intervention on the patients’ diagnostic-therapeutic pathway, informed consent was not required.

### Data collection and measuring tools

The Delay Tool we used was developed by Carey et al. [5], who conducted an observational study at an American university-affiliated tertiary care hospital, with the aim to detect, quantify, and characterise delays that unnecessarily prolonged hospitalisation. It comprises two separate parts (see Additional file 1). The first contains questions aiming to determine whether a hospitalised patient’s clinical status was compatible with discharge. If this is the case, the second part requires to identify the factors that may have contributed to the delay, distinguishing between medical and nonmedical causes. The tool thus allows to identify those patients whose hospital stay was unnecessary, i.e. patients who had no symptoms, signs, or likely diagnoses placing them at high risk for immediate morbidity or mortality, or who had 1 or more of these risks but there was no anticipated risk reduction from hospitalisation. The tool also enables to determine the reasons for failure to discharge, and to gather information on patient age, sex, hospitalisation ward, and date of admission. The Charlson Comorbidity Index [22] was derived from ICD9-CM diagnostic and procedural codes available in administrative datasets. The use of such an indicator ensures a more precise control for casemix, and works as a proxy for severity at patient level: the higher the value, the greater the severity.

Information was collected for both arms by specifically trained personnel (five senior physicians: one Director TM, and four experienced collaborators AN, EP, BP, TS), operating in a ward which did not participate in the study. Training consisted in a half-day seminar, during which TM introduced the project and provided instructions on the use of the tool, followed by a 2-day implementation in the physicians’ own wards and discussion of encountered problems. The analysis included all patients present on the participating wards during one of 12 randomly selected index days (one for each month of data collection). A monthly data collection pattern was considered adequate to ensure that any seasonal variations in organization, patient flow, etc. would be taken into account. Patients admitted or discharged on the index days, and patients with LOS >90 days were excluded. Gathered information was relative to the day preceding the index day, and derived from clinical documentation; healthcare staff was interviewed only if clarifications were needed. During data collection, all five physicians were present in the ward simultaneously, and controversial cases were discussed until agreement was reached.

The monthly reports containing physician length of stay profiles were compiled using hospital administrative data. They contained, for each physician, the number of patients discharged in the month, along with relative mean, observed, and expected LOS. The names of all physicians except that of the recipient were hidden, to ensure anonymity (see Additional file 2). The hospital’s database of discharge summaries, used in this study, is periodically validated against clinical records by trained, external personnel, in accordance with regulations established by the Region [14].

### Sample size

To define the size and test feasibility of the investigation, a pilot study was conducted at one nonparticipating centre (the Neurology Unit of the University Hospital of Parma) in one day. This preliminary investigation allowed to estimate the baseline value of the main endpoint, 50% of patient-days judged compatible with discharge, as well as the proportion of ineligible cases, 9.4% (3/32).

As the primary endpoint was measured over a one-year period, and being 350/day the mean number of patients present in the 12 participating wards, it was estimated that overall approximately 4000 patient-days should be investigated, of which 10% would be ineligible.

We defined an expected difference of 10% (from 50% to 40%), slightly smaller than that reported by Lagoe et al. [8], considering the probable contamination implying an underestimation of the effect.

Intracluster Correlation Coefficient (ICC) was not taken into account when calculating the sample size, because at the time of protocol development no study was available providing an estimate for variance inflation as a result of clustering. Without ICC, a size of 834 had been estimated, assuming a power of 80% at a two-sided significant level of 0.05. However, starting from 4000 patient-days estimated in one year, and accounting for the clustering effect with the mean cluster size of 300, an ICC of 0.02 was obtained, and considered plausible [23,24].

### Statistical analysis

We summarised the baseline characteristics of the clusters using mean number of patient-day (± standard deviation) and the baseline characteristics of the subjects using frequencies (and relative percentages) for categorical variables, and median (with relative interquartile ranges) for continuous variables. We used cluster specific methods because wards, rather than patient-days, were randomised. To account for the clustering of patient-days from the same ward, we used logistic regression with generalized linear mixed model (PROC GENMOD, SAS version release 8.2; SAS Institute, Cary, NC) in the analyses. The ward was considered the unit of cluster for patient-day connected with a specific ward. To control for differences in patient-day and ward characteristics among intervention and control arms, we adjusted for variability both between clusters (wards) and within a cluster (patients within the same ward), and controlled for patient and ward covariates. All analyses were intention-to-treat, and the effects are presented as odds ratios (or incidence rate ratios) and 95% confidence intervals. To assess the model’s goodness-of-fit, the scaled Pearson’s chi-square statistic was used, comparing deviance with its degrees of freedom. The closer the ratio between the two values is to 1, the better the model’s fit. We assessed multicollinearity by examining tolerance and the Variance Inflation Factor (VIF).

## Results

### Study clusters and subjects

**Figure 1:**
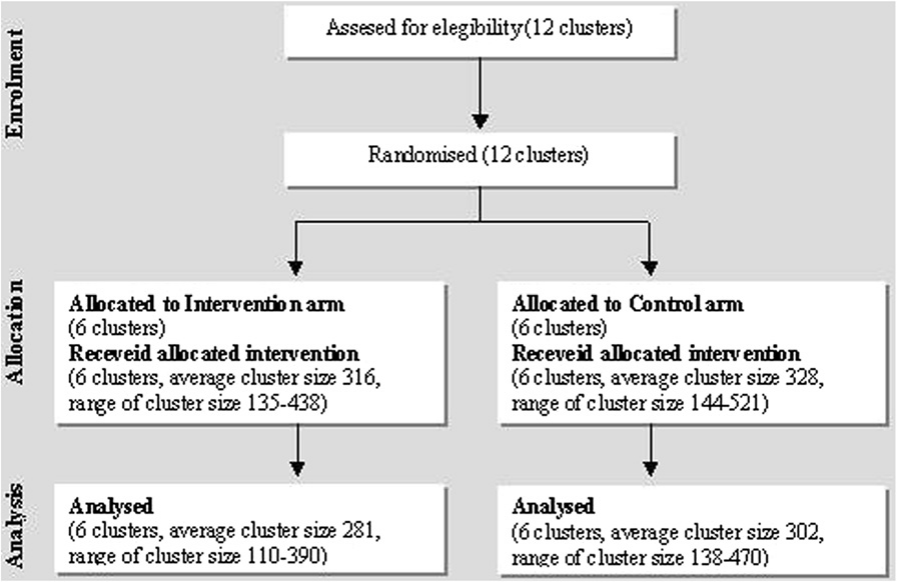
**Flow diagram of the study progress of clusters and subjects.** The Figure represents the study progress based on the CONSORT flowchart.

Figure 1 depicts the study progress based on the CONSORT flowchart.

Units in the three ward types were equally assigned by stratified randomisation to the two arms, which were also similar with respect to the number of beds (178 intervention arm vs 173 control arm), and number of eligible patient-days (Figure 1).

Some differences between the two arms were observed concerning patient characteristics (Table 1): patients in the control arm exhibited a higher degree of comorbidity and were more frequently hospitalised in a long-term care unit. However, such factors were controlled for through multivariate analysis.

**Table 1:** Baseline characteristics of subjects and clusters

		**Intervention arm**	**Control arm**
**Variable**		**(N = 1688)**	**(N = 1810)**
Cluster *			
Age, no./cluster †			
	<=82	140 ± 70	149 ± 89
	>82	141 ± 79	152 ± 94
Female, no./cluster		154 ± 69	166 ± 77
Charlson Comorbidity Index, no./cluster ‡			
	0	52 ± 28	37 ± 16
	1+	228 ± 78	263 ± 116
Ward type, no./cluster ∥			
	Geriatrics unit	361 ± 0	377 ± 0
	Long-term care unit	261 ± 0	363 ± 0
	General Medicine unit	267 ± 125	268 ± 142
**Subjects**			
Age in years - Median (IQR) §		82 (74–87)	82 (75–88)
Female - no (%)		924 (55)	996 (55)
Charlson Comorbidity Index - no (%)¶			
	0	312 (18)	223 (12)
	1	620 (37)	597 (33)
	2	520 (31)	612 (34)
	3	184 (11)	305 (17)
	4	42 (3)	57 (3)
	5	5 (0)	8 (0)
	6	0 (0)	0 (0)
	N.a.	5 (0)	8 (0)
Ward type - no (%)			
	Geriatrics unit	361 (21)	377 (21)
	Long-term care unit	261 (16)	363 (20)
	General Medicine unit	1066 (63)	1070 (59)

*Plus-minus values are means +/− SD.

†Age in Cluster statistics recoded in two classes, based on subjects’ median age (82).

‡Charlson Index in Cluster statistics recoded in two classes: 0 (no comorbidities).

and 1+ (with comorbidities).

∥Geriatrics and Long-term care ward types have 1 unit in both arms: then SD=0.

§IQR = InterQuartile Range.

¶Percentages may not add up to 100 due to rounding.

During the 12-month investigation, data on 3862 patient-days were collected, 3498 of which were eligible (91%), 1688 intervention arm (49%) and 1810 control arm (52%). 55% of cases (1935/3498) was judged to be compatible with discharge.

### Outcomes and estimation

**Figure 2:**
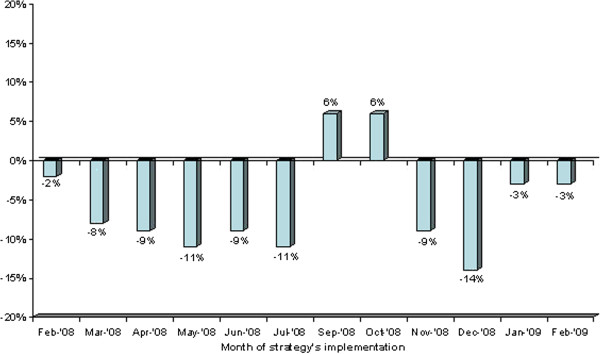
**Differences in percentage of days compatible with discharge.** The rectangular bars represent the differences between intervention and control arms recorded during the months of strategy’s implementation. Percentages of days compatible with discharge in each arm are available in Additional file 3.

During the 12 months of data collection, patient-days judged to be compatible with discharge were 889/1688 (52.7%) intervention arm vs 1046/1810 (57.8%) control arm (OR=0.813; 95% CI, 0.711 to 0.929; P=0.0023). The analysis of the difference between the two arms during each month (Figure 2) showed that the greatest improvement (−8% -14%) occurred from the 2nd through the 6th months and in the 9th and 10th months, with an inverse trend in the 7th and 8th months of implementation.

As shown in Table 2, delays were due to problems with activities under medical staff control for 87% of patient-days (1682/1935) and to nonmedical causes, i.e. activities under social work or case management control, for 13% of patient-days (253/1935). Among the former, the most frequent reason was the “delay in test performance” (34%, 565/1682), followed by presence in the ward “without any apparent reason” (32%, 541/1682).

**Table 2:** Reasons for excessive length of stay

	**Intervention arm**	**Control arm**	**Tot**	**%**		
**Clinical status compatible with discharge**	**889**	**1046**	**1935/3498**	**55%**		
Problem with activities under medical Staff control	787	895	1682/1935	87%		
Problem with activities under social work or case management control	102	151	253/1935	13%		
**Problem with activities under medical Staff control**						
**Reason**	**Intervention arm (N=787)**		**Control arm (N=895)**		**Tot (N=1682)**	**%**
Without any apparent reason	205	**26%**	336	**38%**	541	32%
Test	285	**36%**	280	**31%**	565	34%
Consultation	57	**7%**	63	**7%**	120	7%
Lab test/result	26	**3%**	29	**3%**	55	3%
Test interpretation	7	**1%**	6	**1%**	13	1%
Surgery	11	**1%**	30	**3%**	41	2%
Transfer to other ward	118	**15%**	101	**11%**	219	13%
i.v. Antibiotic therapy	57	**7%**	34	**4%**	91	5%
Other	21	**3%**	16	**2%**	37	2%
**Problem with activities under social work or case management control**						
**Reason**	**Intervention arm (N=102)**		**Control arm (N=151)**		**Tot (N=253)**	**%**
Waiting to be transferred to	57	**56%**	91	**60%**	148	58%
Home care services	21	**21%**	43	**28%**	64	25%
Transportation	1	**1%**	0	**0%**	1	0%
Other	23	**22%**	17	**11%**	40	16%

Table 3 displays data analysis with an adjusted logistic model. The first part presents multivariate analysis relating to the assessment of the primary end point. Results confirmed that the intervention arm exhibited a lower percentage of patients with hospital stay compatible with discharge, equal to −16%, and that the difference was statistically significant (OR=0.841; 95% CI, 0.735 to 0.963; P=0.012). Among the considered variables, only ward type and severity had an effect on outcome; specifically, being hospitalised in a general medicine and geriatrics unit had a protective effect, compared to long-term care.

**Table 3:** Multivariate models

***Strategy’s effectiveness (N=3498)*** *			
**Variable**	**OR**	**95% CI**	***P***
Intervention	0.841	0.735 - 0.963	0.01
Age	1.004	0.998 - 1.010	0.18
Sex	0.921	0.803 - 1.057	0.24
General Medicine Unit	0.604	0.500 - 0.730	<0.001
Geriatrics Unit	0.739	0.592 - 0.924	0.008
Charlson	1.079	1.009 - 1.155	0.02
**Follow-up (N=248) at 1 year** *			
**Variable**	**OR**	**95% CI**	***P***
Intervention	0.818	0.476 - 1.405	0.47
Age	0.998	0.976 - 1.020	0.83
Sex	0.877	0.514 - 1.494	0.63
General Medicine Unit	0.440	0.162 - 1.195	0.11
Geriatrics Unit	0.575	0.183 - 1.808	0.34
Charlson	1.324	0.997 - 1.759	0.05
***Overall LOS (*N=3498*)*** †			
**Variable**	**IRR**	**95% CI**	***P***
Intervention	0.829	0.718 - 0.958	0.01
Age	0.982	0.963 - 1.001	0.07
Sex	0.939	0.758 - 1.163	0.57
General Medicine Unit	0.221	0.174 - 0.282	<0.001
Geriatrics Unit	0.293	0.224 - 0.383	< 0.001
Charlson	1.020	0.958 - 1.087	0.53
**Reason: “ Without any apparent reason ” (*N=1682*)** * ‡			
**Variable**	**OR**	**95% CI**	***P***
Intervention	0.672	0.539 - 0.837	0.001
Age	1.017	1.006 - 1.028	0.003
Sex	0.804	0.644 - 1.005	0.06
General Medicine Unit	0.253	0.188 - 0.341	<0.001
Geriatrics Unit	0.134	0.094 - 0.193	<0.001
Charlson	1.059	0.950 - 1.181	0.30

* Estimates of the relative rate (OR -  Odds Ratio) were obtained from multivariate logistic model.

† Estimates of the relative rate (IRR  -  Incident Rate Ratio) were obtained from multivariate Poisson model.

‡ The number of observations is smaller because the estimates are based on the subset of patient - days showing delays due to problems with activities under medical staff control.

Follow-up at 1 year regarding the percentages of patient-days judged to be compatible with discharge highlighted a statistically non-significant difference between the two arms (OR=0.818; 95% CI, 0.476 to 1.405; P=0.47).

The strategy’s positive effect was also observed on overall hospital days (secondary objective) for patients under study during the index days, 54,221 in the intervention arm (median 18) and 75,429 in the control arm (median 24); specifically, as shown in the third part of Table 3, the adjusted count model confirmed a reduction of 17% of hospital days in the intervention arm (IRR=0.829; 95% CI, 0.718 to 0.958; P=0.011), and the “protective” effect of being hospitalised in a general medicine and geriatrics unit.

Among the types of reasons explaining patient-days compatible with discharge, the most frequent, and most unevenly distributed between the two arms, was “without any apparent reason”; to verify the third secondary objective, multivariate analysis was performed (last part of Table 3) which confirmed the strategy’s positive effect on this cause of delay, with a 33% statistically significant reduction in patient-days (OR=0.672; 95% CI, 0.539 to 0.837; P=0.001).

To investigate whether reducing hospital days may have a negative effect on the quality of hospital care, we analysed 30-day readmission and mortality rates for all eligible patients (N=3498) during the year of implementation. No statistically significant difference was detected between the two arms. Readmission rate in the intervention arm was 64/1688 (3.8%), vs 83/1810 (4.6%) in the control arm (2-Tail Fisher’s Exact Test p=0.273). Mortality rate in the intervention arm was 53/1688 (3.1%), vs 39/1810 (2.2%) in the control arm (2-Tail Fisher’s Exact Test p=0.073).

## Discussion

In this randomised trial we observe a significant effect of the strategy on the primary outcome, in terms of a 16% reduction in avoidable hospital days. Specifically, in six medical wards of a large Italian teaching hospital, the strategy, involving physician direct accountability, proved particularly beneficial for decreasing the days of stay during which patients were present on the ward “without any apparent reason”. It was expected that improvement would occur gradually, requiring organisational adjustments and a cultural change; instead, the observed effect was immediately evident, demonstrating that sensitizing clinicians can be enough to achieve at least some degree of reduction of hospital days, without introducing complex interventions.

To our knowledge, this is the first randomised, controlled trial demonstrating the effectiveness of a multicomponent strategy aimed to reduce unnecessary days of hospitalization. No systematic review demonstrating the efficacy of interventions has been published. Two Cochrane reviews have analysed the impact of specific interventions on various aspects of care, including LOS. One, comprising 21 RCTs, assessed the effectiveness of discharge planning for patients moving from hospital; concerning length of stay, a small, significant reduction was found for patients allocated to discharge planning [25]. Another review, assessing the effect of clinical pathways on professional practice, patient outcomes, length of stay and hospital costs, including 13 RCTs, concluded that care pathways did lead to significant shortening of LOS, particularly for invasive procedures, however substantial heterogeneity between studies was reported [26].

A study exhibiting characteristics comparable to our trial is a large, observational research conducted in four New York hospitals [8], which obtained a 12.2% reduction in LOS over a 3-year period, in line with our findings. It should be noted that compared to the intervention of Lagoe et al., our strategy is less complex, and does not foresee interference from the project team, making professionals responsible for identifying and implementing appropriate corrective measures.

Intervention assessment over a 1-year time frame had two main advantages: firstly, it enabled us to realise the real potential of the strategy, which would have seemed extremely effective if only the first few months of implementation had been analysed. Secondly, it was possible to observe that the effect was neither gradual nor uniform. Concerning this aspect, we cannot rule out that external factors may have somehow influenced results. In particular, the lack of a statistically significant measurable impact in the long-term might partly be due to a Hawthorne effect, i.e. a positive effect caused by participants’ awareness of being observed and monitored, which however doesn’t last with time.

A more modest reduction of hospital days compatible with discharge was also observed in the control arm, which may be explained by a contamination effect, occurring in spite of the measures taken to ensure blinding. In fact, because data gathering was also performed in the wards of the control arm, attention to this problem is likely to have been enhanced in these units as well. This may have diluted the strategy’s effectiveness, although we believe this effect to be small, as wards in the control arm did not receive feedback on which to base improvement interventions.

Our investigation has some limitations. First, results may not be generalisable to other settings because the study was performed in only one university tertiary care hospital, with a high proportion of elderly patients, enjoying an efficient network of community services. This is reflected in the frequency of hospital days judged to be unnecessary found in our study, where only a small percentage (7%) was due to problems under social work or case management control, unlike what reported by Selker [4] and Carey [5] (13%-17%). Lack of comparison due to the monocentric design also does not allow to rule out confounders, therefore transfer of the model to other settings should be done with caution, and preceded by accurate context analysis. Second, it should be noted that the reliability and validity of the instrument used to categorise delays in care have not been established, also because of the lack of a gold standard. Lastly, this study does not assess the impact of the strategy in terms of resource utilisation, a particularly relevant issue in the current difficult global economic situation.

## Conclusions

The results of this study indicate that a strategy involving physician direct accountability can substantially reduce unnecessary, avoidable hospital days, without the need to implement complex interventions. Patients do not appear to have been adversely affected by early discharge, as suggested by the lack of variation in hospital readmission and 30-day mortality. In the light of our experience, we would recommend that hospitals intending to apply such a strategy first perform context analysis, to determine the relevance of the problem and the main causes for unnecessary, avoidable hospital days. In fact, this strategy, focused on physician accountability, will be most beneficial when delays mainly lie in problems with activities under medical staff control. As shown in the literature, unnecessary prolongation of hospital stay can cause harm to patients, and must therefore be prevented.

## Abbreviations

LOS: Length of stay; DRG: Diagnosis-Related Groups; CONSORT: CONsolidated Standards Of Reporting Trials; SQUIRE: Standards for QUality Improvement Reporting Excellence; ICC: Intracluster Correlation Coefficient; VIF: Variance Inflation Factor.

## Competing interests

The authors declare that they have no competing interests.

## Authors’ contributions

CC and FD were involved in the study concept and design. BM, LBo, TM, AN, EP, BP and TS were responsible for data acquisition. CC, EI and LBr were in charge of analysis, interpretation of data and statistical analysis. CC and FD drafted the manuscript. All authors critically revised the manuscript for important intellectual content. LBo was responsible for obtaining funding. CC and BM provided administrative, technical, and material support. CC and TM were study supervisors. All authors had full access to all of the data (including statistical reports and tables) in the study and can take responsibility for the integrity of the data and the accuracy of the data analysis. All authors read and approved the final manuscript.

## Pre-publication history

The pre-publication history for this paper can be accessed here:

http://www.biomedcentral.com/1472-6963/13/14/prepub

## Supplementary Material

Additional file 1 **Delay Tool.** Description: tool used to identify hospital days compatible with discharge and determine the reasons delaying discharge. Click here for file

Additional file 2 **Physician monthly reports.** Description: Length of Stay Profile and List of patients who were classified to be present on the ward although their clinical status was considered compatible with discharge. Click here for file

Additional file 3 **Percentages of days compatible with discharge per arm.**Description: Percentages of days compatible with discharge in intervention and control arms, during each month of the strategy ’s implementation. Click here for file
