# Supplementary material for: Reducing unnecessary hospital days to improve quality of care through physician accountability: a cluster randomised trial
Source: BMC Health Serv Res. 2013 Jan 10;13:14. doi: 10.1186/1472-6963-13-14 (PMC3577481; doi:10.1186/1472-6963-13-14)
Supplement: Additional file 1 — Delay Tool. Description: tool used to identify hospital days compatible with discharge and determine the reasons delaying discharge. [file 1472-6963-13-14-S1.pdf]

SERVIZIO SANITARIO REGIONALE
EMILIA-ROMAGNA
Azienda Ospedaliero - Universitaria di Parma

Programma di Ricerca Regione-Università 2007-2009

Area 2

Research for Clinical Governance

Unnecessary, avoidable lengths of stay: a strategy for clinician empowerment and effectiveness evaluation

Instructions:

1. Data collection concerns all patients present on an index day, randomly identified on a monthly basis

2. Gathered information is relative to the day preceding the index day, and must be based on objective data contained in clinical documentation, to enable reproducibility/testing of judgement

3. Patients who were discharged, admitted, or out on leave during the index day are excluded; in this case one of the options in the upper section of the first page should be selected, stop data collection

4. If the patient is not dimissible, select the corresponding option, stop data collection

5. If the patient's clinical status is compatible with discharge, continue to page 2

6. If a problem with activities under medical staff control is detected (test, procedure,...), continue to step 1

7. If a problem with activities under social work or case management control occurred, continue to step 2

Data collector ______________________

Patient ID number ______________ Sheet number ______________

Last name and first name ________________________ Date of survey ______________ dd/mm/yyyy

Date of hospital admission ______________ dd/mm/yyyy Date of ward admission ______________ dd/mm/yyyy

Date of last hospital admission ______________ dd/mm/yyyy

Long-Term Care Code ☐ No ☐ Yes Date of request ______________ dd/mm/yyyy

The patient was on leave yesterday? ☐ No ☐ Yes

☐ **Patient discharged today**
☐ **Patient admitted today**
☐ **Patient on leave today**

} **Excluded from survey**

Patient clinical condition is not compatible with discharge:

☐ *Presence of symptoms, signs, or likely diagnoses that placed the patient at high risk for immediate morbidity or mortality (stop)*

☐ *No symptoms, signs, or likely diagnoses that place the patient at high risk for immediate morbidity or mortality are present, but hospital stay is necessary because*

____ *(stop)*

Example:

1. 70 year old woman with ischemic heart disease and bedridden hypokinetic syndrome is undergoing FKT to recover verticality and ambulation.

Patient clinical condition is compatible with discharge, because:

☐ *No symptoms, signs, or likely diagnoses that placed the patient at high risk for immediate morbidity or mortality are present (continue)*

Example:

1. 84 yo female with pneumonia on PO antibiotics in whom oxygenation has returned to baseline; awaiting nursing home placement

☐ *Symptoms, signs, or likely diagnoses that place the patient at high risk for immediate morbidity or mortality are present but there is no anticipated risk reduction from hospitalization (continue)*

Examples:

1. Cancer patient receiving palliative care

2. 55 yo male with episodic chest pain and shortness of breath prior to admission, no symptoms in hospital, awaiting stress test to rule out ischemia).

Was there a problem with activities under medical Staff control (i.e. test, procedure…)?

☐ No (go to step Step 2) ☐ Yes (continue)

Step 1

Reason:

☐ Without any apparent reason

Delay in performance of: (Pick one)

☐ Test (RX, CT, Echo, etc)	Specify:	____________
	Date of request:	___/___/___ dd/mm/yyyy
☐ Consultation	Specify:	____________
	Date of request:	___/___/___ dd/mm/yyyy
☐ Lab test/result	Specify:	____________
	Date of request:	___/___/___ dd/mm/yyyy
☐ Test interpretation	Specify:	____________
	Date of request:	___/___/___ dd/mm/yyyy
☐ Surgery	Specify:	____________
	Date of request:	___/___/___ dd/mm/yyyy
☐ Transfer to other ward	Specify:	____________
	Date of request:	___/___/___ dd/mm/yyyy
☐ i.v. Antibiotic therapy	Specify:	____________
	Date of request:	___/___/___ dd/mm/yyyy
☐ Other	Specify:	____________
	Date of request:	___/___/___ dd/mm/yyyy

The intervention was not done as outpatient because (Pick one):

☐ Need to rule out life-threatening disease before discharge

☐ Concern about timely outpatient follow-up

☐ Concern about patient compliance

☐ No concern, just convenience (patient was here so let's get the intervention while s/he is here)

☐ Patient/family demand/expectation/preference

☐ Clinician's "courtesy decision" (commonly accepted practice, friendly relations)

☐ Other (Specify): ____________

Has the difficult discharge procedure been activated?

☐ No ☐ Yes Date ___/___/___

Step 2

Was there a problem with activities under social work or case management control?

- [ ] No, everything was ready for d/c
- [ ] Yes (continue)

Problems linked to:

Waiting to be transferred to:

- [ ] Hospice — Date of request: ____/____/____ (dd/mm/yyyy)
- [ ] Specialized facility — Date of request: ____/____/____ (dd/mm/yyyy)
- [ ] Residential care — Date of request: ____/____/____ (dd/mm/yyyy)
- [ ] Nursing home — Date of request: ____/____/____ (dd/mm/yyyy)
- [ ] Other ____________ — Date of request: ____/____/____ (dd/mm/yyyy)

Home health:

- [ ] Lacking or absent home social services
- [ ] Lacking or absent home nursing services
- [ ] Architectural barriers
- [ ] Waiting for assistance devices
- [ ] Altro ____________

Transportation:

- [ ] No ride
- [ ] Ambulance issue

Specify:

- [ ] Too late for ambulance
- [ ] Not ordered in time
- [ ] No transportation for dialysis
- [ ] Other ____________

Was there a complicating factor?

- [ ] Living outside the Region
- [ ] Foreigners without stable residence
- [ ] Communication with family
- [ ] Financial problems
- [ ] Social issues (living alone)
- [ ] Other ____________
