# Supplementary material for: Reducing unnecessary hospital days to improve quality of care through physician accountability: a cluster randomised trial
Source: BMC Health Serv Res. 2013 Jan 10;13:14. doi: 10.1186/1472-6963-13-14 (PMC3577481; doi:10.1186/1472-6963-13-14)
Supplement: Additional file 2 — Physician monthly reports. Description: Length of Stay Profile and List of patients who were classified to be present on the ward although their clinical status was considered compatible with discharge. [file 1472-6963-13-14-S2.pdf]

Report

Physician Length of Stay Profile

Physician No/Name	Number of Discharges	Over all lenght of stay	Of which discharges over 3 months	Of which discharges over expected mean		Duration of observed LOS	Duration of expected LOS	Difference between observed and expected LOS
				N°	%			
XXXX-1								
XXXX-2								
XXXX-3								
total								

Note: XXXX Ward Code

Report

List of patients who, through data collection performed on the index day with the Delay Tool (see data collection), were classified to be present on the ward although their clinical status was considered compatible with discharge

Patient ID number	Last name and first name	Date of survey	Date of hospital admission	Date of ward admission	Ward	Reason of delay	Comments
