# Supplementary figures and images for: Reducing unnecessary hospital days to improve quality of care through physician accountability: a cluster randomised trial
Source: BMC Health Serv Res. 2013 Jan 10;13:14. doi: 10.1186/1472-6963-13-14 (PMC3577481; doi:10.1186/1472-6963-13-14)

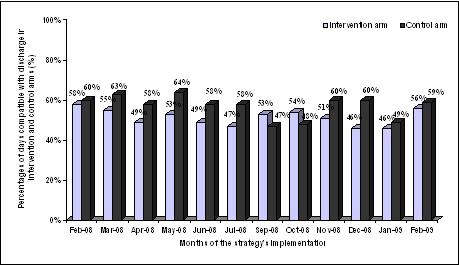

Supplement: Additional file 3 — Percentages of days compatible with discharge per arm.Description: Percentages of days compatible with discharge in intervention and control arms, during each month of the strategy ’s implementation. [file 1472-6963-13-14-S3.jpeg]
